# Standard b-value versus low b-value diffusion-weighted MRI in renal cell carcinoma: a systematic review and meta-analysis

**DOI:** 10.1186/1471-2407-14-843

**Published:** 2014-11-18

**Authors:** Yanlong Tang, Yue Zhou, Wei Du, Ning Liu, Chengzhi Zhang, Tianzhao Ouyang, Jinbo Hu

**Affiliations:** Department of Radiology, the Affiliated Hospital of Dali University, Yunnan, 671000 China; Department of Histology and Embryology, Dali Medical University, Yunnan, 671000 China

**Keywords:** Renal cell carcinoma, RCC, Diffusion-weighted MRI, DW-MRI, b-value

## Abstract

**Background:**

We sought to determine the comparative diagnostic performance of standard b-value (800–1000 s/mm^2^) versus low b-value (400–500 s/mm^2^) diffusion-weighted magnetic resonance imaging (DW-MRI) in the detection of renal cell carcinoma (RCC).

**Method:**

After a systematic review of the available literature, studies were included that reported b-values, used a histopathological reference standard, and allowed construction of 2 × 2 contingency tables for detection of RCC lesions using DW-MRI. In addition, a summary receiver operating characteristic (SROC) analysis was performed.

**Results:**

Four articles that complied with all inclusion and exclusion criteria were selected for data extraction and analysis (n = 248 lesions in 266 patients). All four studies were high quality. Standard b-value DW-MRI displayed a pooled sensitivity of 0.59 (95% confidence interval (CI): 0.51-0.67) and a pooled specificity of 0.50 (95% CI: 0.30-0.70), while low b-value DW-MRI displayed a pooled sensitivity of 0.58 (95% CI: 0.48-0.63) and a pooled specificity of 0.23 (95% CI: 0.09-0.44). The SROC curve of standard b-value DW-MRI displayed an AUC of 0.61 and a Q*index of 0.59, while the SROC curve of low b-value DW-MRI displayed an AUC of 0.68 and a Q*index of 0.64.

**Conclusion:**

Standard b-value DW-MRI showed a superior specificity but an approximately equivalent sensitivity to low b-value DW-MRI in detecting RCC lesions in the kidney. However, low b-value DW-MRI displayed an overall superior diagnostic accuracy over standard b-value DW-MRI.

## Background

Renal cell carcinoma (RCC) is the most common form of adult renal cancer, accounting for 85-90% of kidney neoplasms and ~3% of adult malignancies [1]. Unfortunately, many RCC tumors are asymptomatic and non-palpable in their early stages; therefore, greater than 50% of RCC tumors are incidentally detected by diagnostic imaging [2]. Due to a paucity of effective screening tests, approximately a third of RCC patients present with metastasis at the time of diagnosis. Moreover, 30-50% of kidney-localized RCC eventually metastasize with a median survival of 10.2 months and a five-year survival rate under 15% [3, 4].

Currently, renal lesions are evaluated using contrast-enhanced computed tomography (CT) and magnetic resonance imaging (MRI). False-negative interpretations occur when imaging necrotic or cystic malignant renal lesions that can be mistakenly interpreted as complex renal cysts due to a lack of enhancement [5, 6]. Moreover, contrast-enhanced studies are typically precluded in patients who have renal impairment or allergies to contrast agents [7]. These clinical limitations have led to the use of other imaging modalities, such as diffusion-weighted MRI (DW-MRI), which provide both qualitative and quantitative tissue characterization without the need for contrast enhancement.

DW-MRI functions by visualizing the random (Brownian) motion of water molecules within tissues [8]. Specifically, motion probing gradients are applied to non-directionally sensitize water molecules in order to determine water movement between diffusion-sensitizing gradient pulses [9]. If water moves substantially between diffusion-sensitizing gradients, the resulting bulk water signal is low; however, if water is restricted from moving between these gradients, the signal is high [9]. The diffusion gradient strength is termed the b-value [s/mm2] and is dependent on the duration and amplitude of the diffusion sensitizing gradient as well as the time between applications of the sensitizing gradient; therefore, in order to increase the b-value during DW-MRI, a greater amplitude of the diffusion-sensitizing gradient is typically applied [9].

Through linear regression, images taken at various b-values can then be used to calculate the apparent diffusion coefficient (ADC) in a particular region of interest. With respect to focal renal lesions, solid malignancies typically display lower ADC values than benign lesions, possibly related to the high cellular density of tumors with intact cell membranes that impedes the Brownian motion of water molecules. One meta-analysis of 17 studies has demonstrated that ADC values can help distinguish between benign and malignant RCC tumors with RCC tumors displaying significantly lower ADC values than benign kidney tissue [8].

Although ADC values of RCC tumors have been well-analyzed by previous studies, no study has yet examined the b-values of DW-MRI with respect to RCC. This is of clinical importance, as factors aside from passive diffusion, such as capillary perfusion, can contribute to decreased signal-to-noise ratio (SNR) in low b-value DW-MRI [10]. On account of this signal decay, low b-value DW-MRI becomes less qualitative and more quantitative, since it must be based on complex ADC calculations. Therefore, as low b-value DW-MRI does not facilitate qualitative detection of malignancies which may adversely affect diagnostic accuracy, the objective of this study was to determine the comparative diagnostic performance of standard b-value (800–1000 s/mm^2^) versus low b-value (400–500 s/mm^2^) DW-MRI in the detection of RCC.

## Methods

### Ethics statement

All data were extracted from previously published studies. We merged these data to perform the meta-analysis as follows.

### Search strategy

A systematic review of the available literature was performed according to the PRISMA (preferred reporting items for systematic reviews and meta-analyses) guidelines [11]. Relevant randomized controlled trials (RCTs) were identified from systematic searches of several major electronic databases (MEDLINE via PubMed, EMBASE, and the Cochrane Central Register of Controlled Trials via Ovid) up to November 2013 with different combinations of the following key words: (“diffusion-weighted” OR “DWI”) AND (“magnetic resonance imaging” OR “MRI”) AND (“ADC” OR “apparent diffusion coefficient”) AND (“renal cell carcinoma” OR “RCC” OR “renal carcinoma” OR “renal cancer” OR “kidney cancer”). Additional relevant articles were obtained by scanning conference summaries and article reference lists identified in the initial searches. An English language restriction was imposed.

### Inclusion and exclusion criteria

Studies were selected for inclusion on the basis of the following criteria: assessing of the diagnostic performance of DW-MRI in evaluating RCC; providing histopathological results; providing b-values and ADC values; presenting sufficient information to calculate the true-positive (TP), false-positive (FP), true-negative (TN), and false-negative (FN) values for construction of 2 × 2 contingency tables. Studies were excluded on the basis of the following criteria: the same study population was assessed in more than one publication (in this case, the publication with the most details and/or the most recent publication date was chosen); the performance assessment of DW-MRI alone could not be extracted; or the articles are reviews, editorials, commentaries, or case reports.

### Study selection and data extraction

The titles and abstracts of studies identified by the search strategy were independently screened by two reviewers, and clearly irrelevant studies were discarded. The full texts were obtained from all articles which met the inclusion criteria. Then, the articles were scanned and the data from these studies were extracted, including: first author's name, year of publication, study design, number of patients per arm, total number of lesions imaged, reference or gold standard (e.g., whole-mount or step-section histopathology, biopsy), coil type (e.g., torso surface phased-array, endorectal, body coil), field strength (e.g., 1.5 T, 3.0 T), b-value, and TP, FP, TN, and FN values for construction of 2 × 2 contingency tables. Disagreements between the two reviewers were resolved by majority opinion after a third reviewer assessed all involved items.

### Quality assessment

The methodological quality of the included studies was assessed by two independent observers using the Quality Assessment of Diagnostic Studies (QUADAS) instrument specifically developed for systematic reviews of diagnostic test accuracy [12].

### Meta-analysis

Data were analyzed using Meta-Disc (version 1.4) software [13, 14]. We pooled the data with the DerSimonian-Laird random effects model (REM) [15–17]. This REM provides more conservative estimates with wider confidence intervals, as it assumes that the meta-analysis includes only a sample of all possible studies [18, 19]. In addition, this REM accounts for both within-study variability (random error) and between-study variability (heterogeneity). We used Chi-square analysis to detect heterogeneity in the summary results.

Each study in the meta-analysis contributed data to form 2 × 2 contingency tables to determine sensitivity and specificity [20, 21]. We then performed a summary receiver operating characteristic (SROC) curve analysis. The SROC displays a study's estimated sensitivity and specificity within the ROC space. A regression curve is then fitted through the distribution of sensitivity and specificity pairs. A shoulder-like curve reveals that the inter-study variability may be due to a threshold effect, while a non-shoulder-like curve indicates that sensitivity and specificity are not correlated [19, 22]. The area under the SROC curve (AUC) demonstrates the trade-off between specificity and sensitivity, showing the overall summary of diagnostic performance with an AUC of 1.0 (100%) indicating a perfectly discriminating test [23]. In addition, we calculated the Q* index – defined by the point where sensitivity equates to specificity on the SROC curve – as a global estimate of diagnostic accuracy to enable comparison of SROC curves with a Q* value of 1.0 indicating 100% sensitivity and 100% specificity [24, 25].

## Results

**Figure 1:**
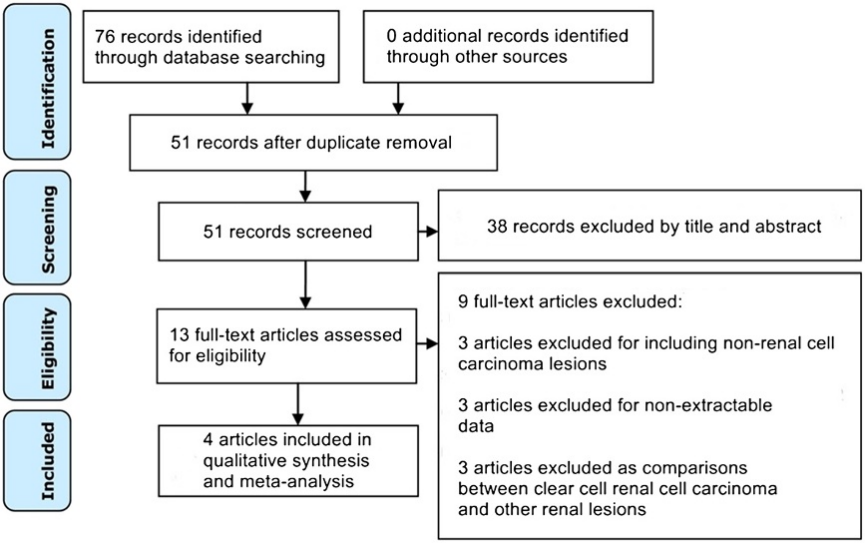
**Flow diagram of study selection.**

After the initial computer search, manual crosschecking of reference lists, and elimination of duplicate records, 51 unique records were identified (Figure 1). Next, the titles and abstracts were reviewed, resulting in 13 eligible full-text articles. After reviewing the 13 full-text articles, we excluded 9 relevant articles for various reasons described in Figure 1. The remaining four articles complied with all inclusion and exclusion criteria and were selected for data extraction and data analysis (Table 1) [26–29]. According to QUADAS assessment, all four studies were of high quality (Table 2).

**Table 1:** **Characteristics of included studies**

Study	Design	Total number of patients	Total number of lesions imaged	Reference standard	Coil type	Field strength (T)	B-value (s/mm ^2^ )
Wang 2010 [25]	Retrospective	83	85	Histopathology	Surface phased-array coil	3.0	500, 800
Rosenkrantz 2010 [24]	Retrospective	57	57	Histopathology	Torso phased-array coil	1.5	400, 800
Chandarana 2012 [28]	Prospective	26	26	Histopathology	Torso phased-array coil	1.5	1000
Goyal 2013 [29]	Retrospective	100	80	Histopathology	Phased-array body coil	1.5	500

**Table 2:** **Methodological quality of included studies**

Item	Wang 2010 [25]	Rosenkrantz 2010 [24]	Chandarana 2012 [28]	Goyal 2013 [29]
Was the spectrum of patients clearly representative of the patients who will receive the test in practice?	Y	Y	Y	Y
Were selection criteria clearly described?	Y	Y	Y	Y
Is the reference standard likely to correctly classify the target condition?	Y	Y	Y	Y
Is the time period between reference standard and index test short enough to be reasonably sure that the target condition did not change between the two tests?	Y	Y	U	U
Did the whole sample or a random selection of the sample receive verification using a reference standard of diagnosis?	Y	Y	Y	N
Did patients receive the same reference standard regardless of the index test result?	Y	Y	Y	Y
Was the reference standard independent of the index test (i.e. the index test did not form part of the reference standard)?	Y	Y	Y	Y
Was the execution of the index test described in sufficient detail to permit replication of the test?	Y	Y	Y	Y
Was the execution of the reference standard described in sufficient detail to permit its replication?	Y	Y	Y	Y
Were the index test results interpreted without knowledge of the results of the reference standard?	Y	Y	Y	Y
Were the reference standard results interpreted without knowledge of the results of the index test?	Y	Y	U	U
Were the same clinical data available when test results were interpreted as would be available when the test is used in practice?	Y	Y	Y	Y
Were missing data on the index test handled correctly?	Y	Y	Y	Y
Were withdrawals from the study explained?	Y	Y	Y	Y

*Abbreviations*: *Y* yes, *N* no, *U* unclear.

A total of 248 lesions in 266 patients were used in this meta-analysis. The reference standard in all four studies was histopathology. The random effects model was used in all cases. The number of publications was sufficient to run the random effects model in all cases.

**Figure 2:**
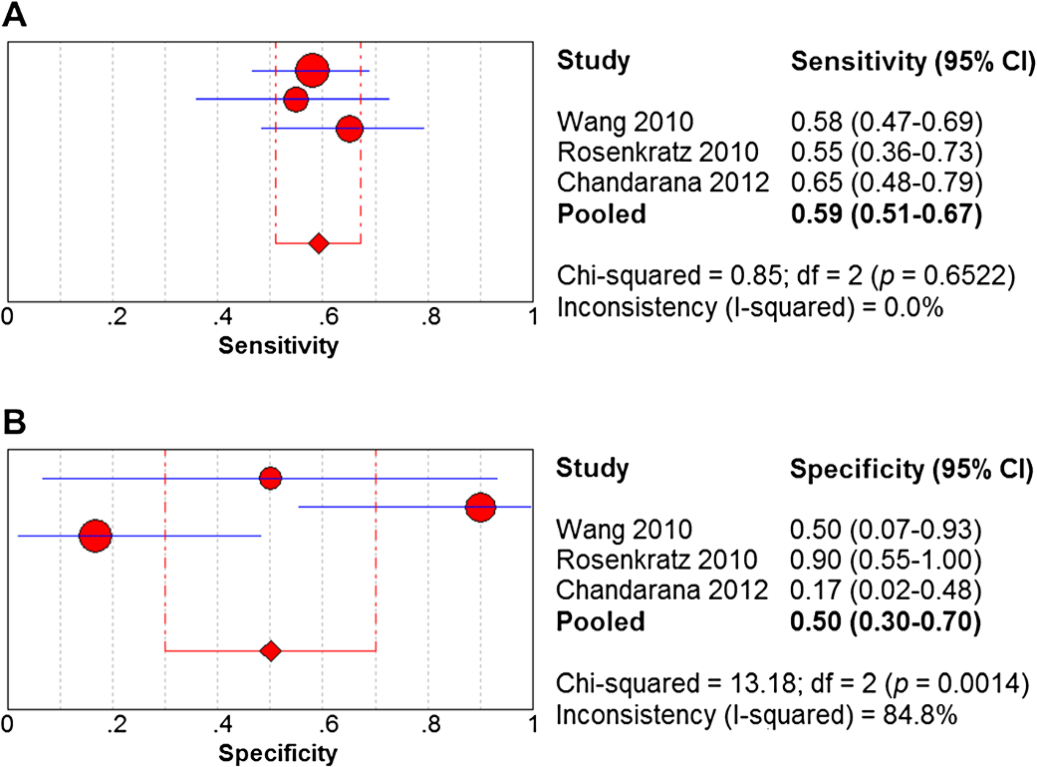
**Forest plots of sensitivity and specificity estimates for standard b-value DW-MRI in detecting renal cell carcinoma.** Point estimates of **(A)** sensitivity and **(B)** specificity from each study are shown as solid red circles. The solid blue lines represent the 95% confidence intervals (CI). Circles are proportional to study size. The pooled estimates are denoted by the red diamonds at the bottom.

**Figure 3:**
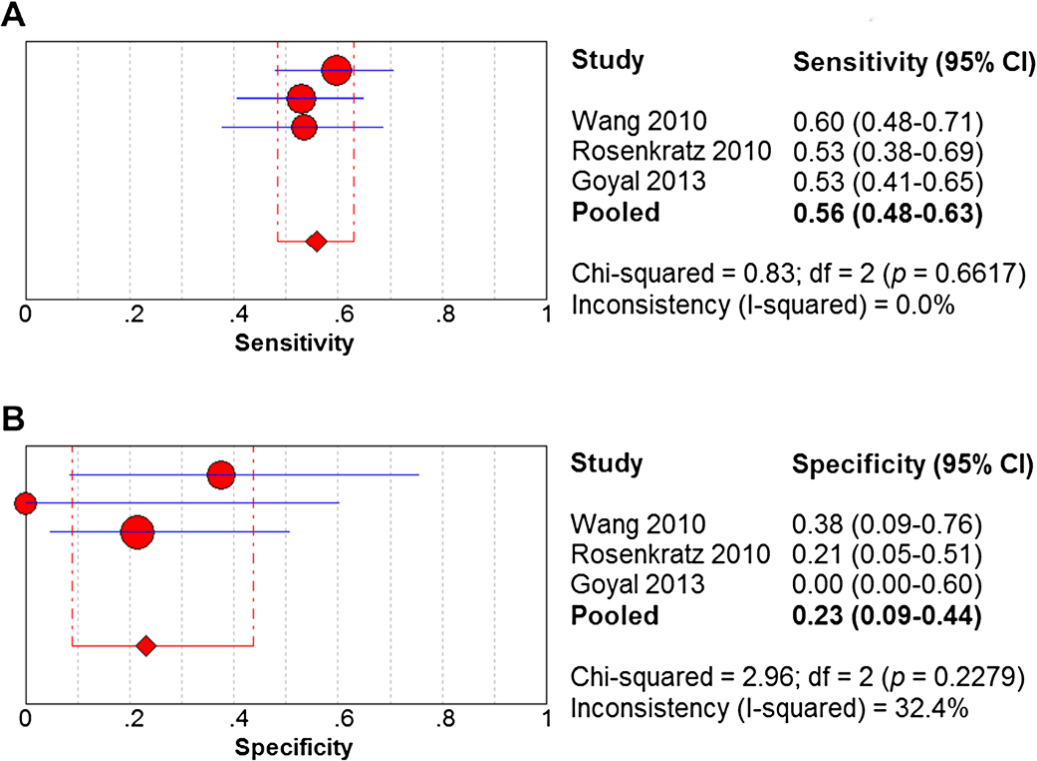
**Forest plots of sensitivity and specificity estimates for low b-value DW-MRI in detecting renal cell carcinoma.** Point estimates of **(A)** sensitivity and **(B)** specificity from each study are shown as solid red circles. The solid blue lines represent the 95% confidence intervals (CI). Circles are proportional to study size. The pooled estimates are denoted by the red diamonds at the bottom.

**Figure 4:**
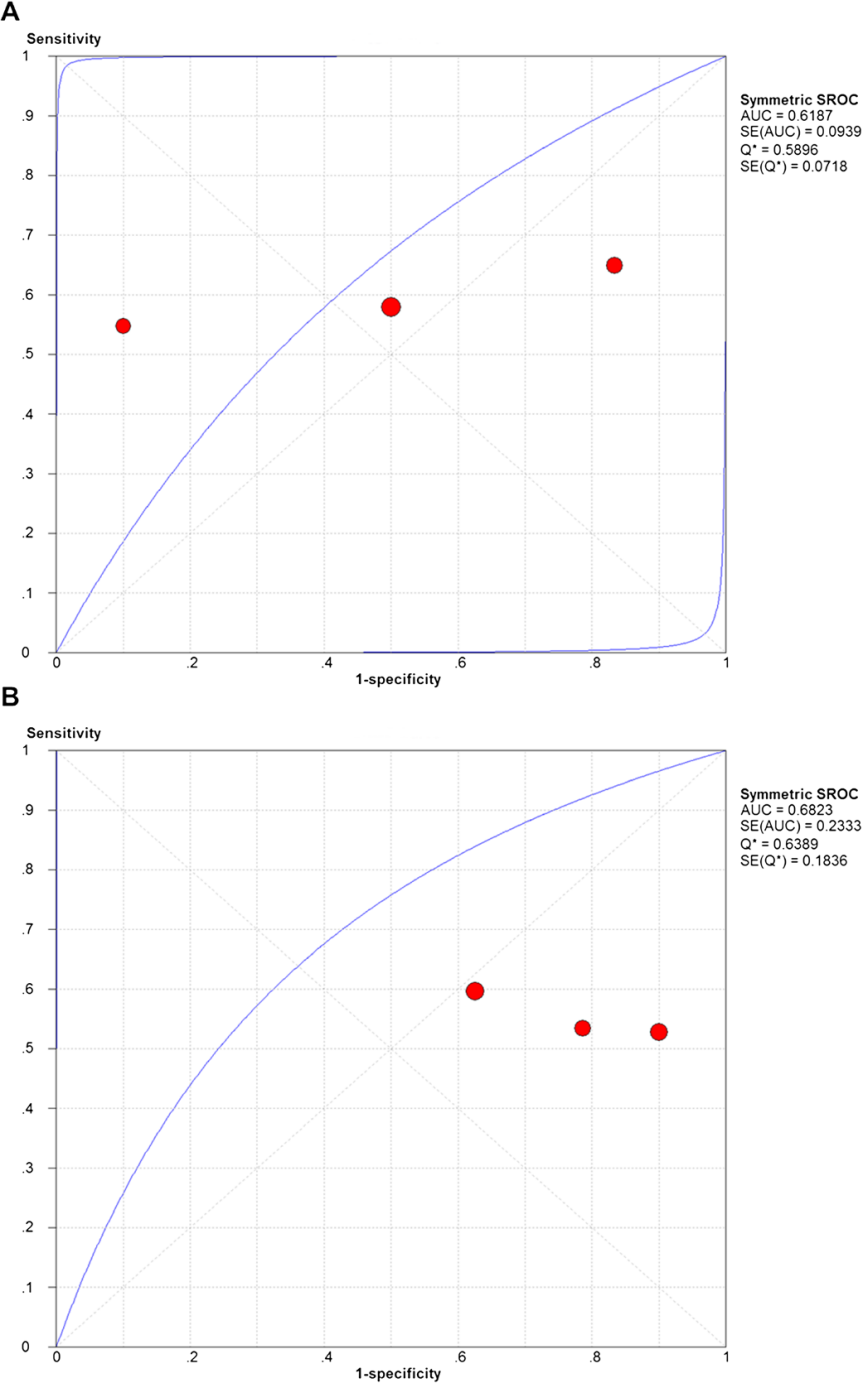
**Summary receiving operating characteristic plot with best-fitting asymmetric curve for standard and low b-value DW-MRI in detecting renal cell carcinoma.** Summary receiving operating characteristic (SROC) plot with best-fitting asymmetric curve for **(A)** standard and **(B)** low b-value DW-MRI. Each solid red circle represents each study in the meta-analysis. The blue curve is the regression line that summarizes the overall diagnostic accuracy. SROC = summary receiver operating characteristic; AUC = area under the curve; SE(AUC) = standard error of AUC; Q* = an index defined by the point on the SROC curve where the sensitivity and specificity are equal, which is the point closest to the top-left corner of the ROC space; SE(Q*) = standard error of Q* index.

Standard b-value (800–1000 s/mm^2^) DW-MRI displayed a sensitivity of 0.59 (95% confidence interval (CI): 0.51-0.67) and a specificity of 0.50 (95% CI: 0.30-0.70) in detecting RCC (Figure 2), while low b-value (400–500 s/mm^2^) DW-MRI displayed a sensitivity of 0.58 (95% CI: 0.48-0.63) and a specificity of 0.23 (95% CI: 0.09-0.44) in detecting RCC (Figure 3). For the standard b-value analysis, the chi-square values for the sensitivity, specificity, positive likelihood ratio, negative likelihood ratio, and diagnostic odds ratio were 0%, 84.8%, 76.5%, 68.9%, and 66.3%, respectively; thus, the heterogeneity in the standard b-value analysis was high. For the low b-value analysis, the chi-square values for the sensitivity, specificity, positive likelihood ratio, negative likelihood ratio, and diagnostic odds ratio were 0%, 32.4%, 15.6%, 0%, and 0%, respectively; thus, the heterogeneity in the low b-value analysis was low. The SROC curve of standard b-value DW-MRI displayed an AUC of 0.61 and a Q*index of 0.59, while the SROC curve of low b-value DW-MRI displayed an AUC of 0.68 and a Q*index of 0.64 (Figure 4).

## Discussion

On account of signal decay, low b-value DW-MRI cannot be qualitative in nature but must be quantitatively based on complex calculations of ADC values [10]. On the other hand, higher b-value DW-MRI typically uses an acquisition method with multiple excitations to improve the SNR and provides better contrast on account of its reflection of more tissue diffusivity and less T2 shinethrough effect [14, 30]. Although the multiple excitations applied in higher b-value DW-MRI can produce increases in motion artifacts, these artifacts are averaged over the multiple excitations by motion-probing gradients and become inconspicuous in the reconstructed images. Thus, with increasing b-values, better qualitative images with a superior SNR are achieved while sacrificing quantitative absolute ADC values that become impossible to calculate on account of signal averaging.

In this study, standard b-value DW-MRI (800–1000 s/mm^2^) showed a superior specificity (0.50 vs. 0.23) but an approximately equivalent sensitivity (0.59 vs. 0.58) to low b-value DW-MRI (400–500 s/mm^2^) in detecting RCC lesions in the kidney (Figures 2, 3). However, low b-value DW-MRI displayed an overall superior diagnostic accuracy over standard b-value DW-MRI as measured by their respective SROC curves (AUC: 0.68 vs. 0.62; Q* index: 0.64 vs. 0.59) in detecting RCC lesions in the kidney (Figure 4). Although this study exclusively focused on the effects of varying b-values on the diagnostic accuracy of detecting RCC lesions in the kidney, two previous studies have examined varying b-values in differentiating malignant from benign renal lesions in the aggregate (i.e., not specifically RCC lesions). In contrast to our findings, Doganay et al. and Erbay et al. collected diffusion data across multiple b-values in patients with various renal mass pathologies and demonstrated that detection of malignant renal lesions improves at b-values of greater than 600 s/mm^2^[31, 32]. These findings suggest that optimal b-values vary across different types of renal lesions; thus, future studies should focus on determining the optimal b-values on a renal tumor-specific basis.

RCC tumors are unique due to the presence of hemosiderin deposits, a phenomenon which has proven useful in their differentiation from other tumor types [32, 33]. According to a recent study by Childs et al., the paramagnetic effect of hemosiderin is likely responsible for in-phase signal intensity losses and T2*-induced intravoxel dephasing commonly observed in RCC lesions [32]. This local magnetic susceptibility-induced intravoxel dephasing is important to DW-MRI of RCC lesions since a greater degree of intravoxel dephasing results in greater loss of signal intensity [31]. This phenomenon may contribute to the limited sensitivity of DW-MRI for the diagnosis of malignant renal masses observed here (i.e., 0.59 for standard b-value DW-MRI and 0.58 for low b-value DW-MRI). Raising the b-value increases the degree of diffusion weighting (i.e., increases the signal loss caused by the diffusion of water molecules along the direction of the applied gradient), which increases the contrast between tissues with different diffusion coefficients while also decreasing the overall signal intensity and SNR [34]. Thus, the underlying loss of signal intensity from hemosiderin-induced intravoxel dephasing combined with the loss of signal intensity from applying a higher b-value may explain why standard b-value DW-MRI displayed an overall inferior diagnostic accuracy over low b-value DW-MRI in detecting RCC lesions here (AUC of 0.62 for standard b-value DW-MRI vs. 0.68 for low b-value DW-MRI).

There also have been numerous studies that have examined the effect of varying b-values on the diagnostic accuracy of detecting malignant lesions in other abdominal tissues. For example, Wu et al. analyzed DW-MRI in combination with conventional MRI and found that a b-value of 1500 s/mm^2^ significantly improved the specificity, but not the sensitivity, in diagnosing upper urinary tract cancer compared to a b-value of 500 s/mm^2^[34]. Koc et al. found that DW-MRI with b-values of 600 s/mm^2^ and higher can better differentiate benign and malignant abdominal and gynecological lesions [33, 35]. Bozcurt et al. analyzed DW-MRI in combination with conventional MRI and found that a b-value of 800 s/mm^2^ increased specificity with no significant affect on sensitivity and accuracy in diagnosing peritoneal tumors compared to a b-value of 400 s/mm^2^[36]. Goshima et al. demonstrated that a b-value of 100 s/mm^2^ possesses a higher sensitivity for malignant hepatocellular carcinoma lesions as compared to higher b-values (i.e., 200, 400, and 800 s/mm^2^) but demonstrated comparable specificities across all b-values [37]. These studies indicate that varying b-values can significantly affect the diagnostic accuracy of DW-MRI's detection of malignant lesions; however, there is no clear trend favoring high or low b-values across different tissue and tumor types. Therefore, further studies are required to determine the optimal b-values on a tissue-specific and tumor-specific basis.

This meta-analysis has several limitations. First, the number of included studies was relatively small. Second, three included studies only included clear cell RCC cases (the most common RCC variant accounting for 70% of cases in surgical series) [38], while one study (Wang 2010) included cases of both clear cell and non-clear cell RCC, which may have adversely affected the meta-analysis. Third, this meta-analysis included negative cases but did not include other types of renal tumors or benign kidney conditions. Thus, the specificity reported here should be considered relative rather than absolute. Fourth, we did not evaluate metastasis here; our sole purpose was to evaluate the diagnostic ability of standard versus low b-value DW-MRI in detecting kidney RCC lesions. Fourth, as no study with a b-value of greater than 1000 s/mm^2^ was included here, further trials in RCC patients are needed to determine whether increasing b-values beyond 1000 s/mm^2^ affects the diagnostic accuracy of detecting RCC lesions in kidney tissue.

## Conclusion

Standard b-value DW-MRI showed a superior specificity but an approximately equivalent sensitivity to low b-value DW-MRI in detecting RCC lesions in the kidney. However, low b-value DW-MRI displayed an overall superior diagnostic accuracy over standard b-value DW-MRI in detecting RCC lesions in the kidney. Further studies that address the limitations discussed herein are needed to support our findings.
